# Gender inequality, women's empowerment, and adolescent birth rates in 363 Latin American cities

**DOI:** 10.1016/j.socscimed.2022.115566

**Published:** 2023-01

**Authors:** Ariela Braverman-Bronstein, Ana F. Ortigoza, Dèsirée Vidaña-Pérez, Tonatiuh Barrientos-Gutiérrez, Laura Baldovino-Chiquillo, Usama Bilal, Amélia Augusta de Lima Friche, Francisco Diez-Canseco, Julie Maslowsky, Alejandra Vives V., Ana V. Diez Roux

**Affiliations:** a Urban Health Collaborative, Dornsife School of Public Health, Drexel University, Philadelphia, USA; b Department of Epidemiology and Biostatistics, Dornsife School of Public Health, Drexel University, Philadelphia, USA; c Center for Survey Research and Evaluation, National Institute of Public Health Cuernavaca, Mexico; d Center for Population Health Research, National Institute of Public Health, Cuernavaca, Mexico; e School of Medicine, Universidad de Los Andes, Bogotá, Colombia; f Observatory for Urban Health in Belo Horizonte, School of Medicine, Federal University of Minas Gerais, Belo Horizonte, Brazil; g CRONICAS Center of Excellence in Chronic Diseases, Universidad Peruana Cayetano Heredia, Lima, Peru; h Center of Excellence in Maternal and Child Health School of Public Health, University of Illinois at Chicago, Chicago, Illinois, USA; i Department of Public Health, School of Medicine / CEDEUS. Pontificia Universidad Católica de Chile. Santiago de Chile, Chile

**Keywords:** Adolescent birth rates, Gender equity, Women empowerment, Urban, Latin America, ABR, Adolescent Birth Rates, GII, Gender Inequality Index, SD, Standard Deviation, LA, Latin America

## Abstract

**Background:**

Gender inequality is high in Latin America (LA). Empowering girls and young women and reducing gender gaps has been proposed as a pathway to reduce adolescent pregnancy. We investigated the associations of urban measures of women's empowerment and gender inequality with adolescent birth rates (ABR) in 366 Latin American cities in nine countries.

**Methods:**

We created a gender inequality index (GII) and three Women Achievement scores reflecting domains of women's empowerment (employment, education, and health care access) using censuses, surveys, and political participation data at city and sub-city levels. We used 3-level negative binomial models (sub-city-city-countries) to assess the association between the GII and scores, with ABR while accounting for other city and sub-city characteristics.

**Results:**

We found within country heterogeneity in gender inequality and women's empowerment measures. The ABR was 4% higher for each 1 standard deviation (1-SD) higher GII (RR 1.04; 95%CI 1.01,1.06), 8% lower for each SD higher autonomy score (RR 0.92; 95%CI 0.86, 0.99), and 12% lower for each SD health care access score (RR 0.88; 95%CI 0.82,0.95) after adjustment for city level population size, population growth, homicide rates, and sub-city population educational attainment and living conditions scores.

**Conclusion:**

Our findings show the key role cities have in reducing ABR through the implementation of strategies that foster women's socioeconomic progress such as education, employment, and health care access.

## Highlights

• Levels of women empowerment and gender inequality vary in Latin American cities.
• Higher city gender inequality is associated with higher adolescent birth rates.
• Higher city women empowerment is associated with lower adolescent birth rates.
• Urban policies targeting gender gaps might help reduce adolescent birth rates.

## 1 Introduction

Latin America (LA) is one of the most unequal regions of the world which affects the lives of millions of adolescent girls (United Nations Development Fund, 2021; UNICEF Latin America and The, 2019). Despite the implementation of policies to promote women's education, improve women's protection, and increase women's rights, adolescent girls and women still face greater disadvantages than their male counterparts (Pan American Health Organization (PAHO) and United Nations Population Fund (UNFPA), 2016). Latin America has undergone a rapid disorganized urbanization with more than 80% of its population living in cities. (Montero, García Editoras) Of urban residents about half are women and 20% is between 14 and 24 years old. (United Nations Youth) Yet, city-level data and research on gender inequality and its implications for women and adolescent girls are currently lacking.

LA has the second-highest adolescent birth rate (ABR) worldwide (60.7 births per 1000 women 15–19 years old), surpassed only by Sub-Saharan Africa (100.5 births per 1000 women 15–19 years old) (United Nations, 2020). Most research has focused on individual-level factors related to reproductive health and education, with less focus on the upstream factors found in the urban environment that may influence cultural and social decisions related to adolescent behavior in LA (Bearinger et al., 2007; Pradhan et al., 2015). A cross-country study of ABR from 1990 to 2012 (including LA and other regions), found that rates declined in countries that experienced greater economic growth and reductions in income inequality, which may be explained by the increase in employment and education opportunities (Santelli et al., 2017). Other evidence from national surveys suggests that lower individual education levels and socioeconomic status are associated with higher ABR (Azevedo et al., 2012), pointing to social and economic conditions as important upstream determinants of ABR.

Gender inequality and the gendered division of labor enabled by the cultural belief that motherhood is the most important female role (and provides a sense of adulthood in young women), reinforced by the lack of educational and work opportunities for women creates conditions in which adolescent pregnancy is accepted and in some instances desired (Azevedo et al., 2012; Estrada et al., 2018; Näslund-Hadley and Binstock, 2010). Also, LA is characterized by a lack of representation of women in government and leadership roles within the region, which reinforces the patriarchal society structure, enables gender discrimination, and further perpetuates the gender inequality cycle (Fraile and Gomez, 2017; Drewry and Garcés-Palacio, 2020). Empowering girls and women in the economic, political, and educational spheres has been associated with better adolescent sexual and reproductive health outcomes (Nkhoma et al., 2020). Women's participation in politics has been shown to impact the development of policies aimed at improving education and working conditions for women, along with reducing economic and social inequality (United Nations D of and Social Affairs, 2015; Rai, 2005). Improving educational, political, and economic opportunities for women could also impact adolescent pregnancy by providing adolescents with more prospects and alternative life-plans thus delaying pregnancy.

A number of policies have targeted known individual-level risk factors for adolescent pregnancy, including comprehensive sex education programs, adolescent friendly health care services, and free access to contraceptive methods (Pozo et al., 2015; Chandra-Mouli et al., 2013). Yet, these policies have not been enough to reduce adolescent birth rates in LA (Caffe et al., 2017). Research on the social determinants of adolescent pregnancy has focused mainly on poverty, education, and other individual-level risk factors (Bearinger et al., 2007; Pradhan et al., 2015; Neal et al., 2018). Multi-country research of adolescent birth rates has generally compared data at a national or subnational level and has encompassed both urban and rural areas. The role of urban environments (including local and city-level factors) may be especially relevant to adolescent pregnancies in highly urbanized LA, given that the majority of the region's population (including a high proportion of youth and adolescents) lives in urban areas (Youth O of the S-GE on. UN-HABITAT and Youth, 2013). Understanding how these factors are associated with ABR could help design more effective interventions at a local level encompassing a broader approach that leverages a broad set of urban policies. To address these gaps, and using harmonized data encompassing country, city, and sub-city characteristics from 363 cities in nine LA countries, we investigated the associations of city-level measures of gender inequality and women's empowerment, proxied by measures of women's employment, education, and health care utilization, with sub-city ABR while adjusting for other city and sub-city level factors.

## 2 Methods

We used data from the *Salud Urbana en America Latina Project* (SALURBAL), which has compiled and harmonized health, social and built-environment data from 371 cities (population ≥100,000 in 2010) in eleven countries of LA (Quistberg et al., 2018). Each city is composed of one or more administrative sub-units (ie, *municipios, comunas, distritos, partidos, delegaciones, cantones or corregimientos*), which we will refer to as sub-cities. This study includes all sub-city units with available data both on births to adolescent mothers aged 15–19 years from 2014 to 2016 and on women's empowerment. We included 1403 sub-cities from 363 cities in nine countries: Argentina, Brazil, Chile, Colombia, Costa Rica, Guatemala, Mexico, Panama, and Peru. The SALURBAL study protocol was approved by the Drexel University Institutional Review Board (IRB) (ID#1612005035) and by appropriate site-specific IRBs.

The outcome of interest was sub-city ABR defined as the total number of live births per 1000 women aged 15 years–19 years. We pooled the number of live births for years 2014–2016 from vital registration data from each country and linked to sub-city level based on the mother's place of residence at the time of birth of the child. We obtained population denominators of women aged 15–19 years living in each sub-city from population projections based on census data (Bilal et al., 2021). All the countries included in our study have a national coverage of birth registration above 90%, suggesting good quality of the data and less risk of underestimating ABR (UNICEF. Birth, 2016).

Our main exposures of interest were gender inequality measured through a Gender Inequality Index (GII), and women's empowerment, proxied by women's achievements (WA scores) in three domains all measured at the city level. While gender inequality and women's empowerment are related constructs, we distinguished inequality from empowerment and considered gender inequality as the position of women in relation to men in education, political representation, and employment; while the empowerment measures refer to the positions women have achieved in autonomy (as proxied by employment and child marriage) education, and health care access.

The GII was designed to capture losses to society in potential human development due to inequality between men and women in the dimensions of health, empowerment (educational and political), and labor market participation. The three women's achievements scores represent achievements in autonomy (increased labor market participation and reduced formal child marriage), education, and health care access. Specific variables were selected after a review of women's empowerment measures and based on data availability at the city level for the countries included in this study. In addition, we assessed associations with the individual indicators used to create these measures to identify specific factors driving the associations of the GII and the WA scores. All indicators were measured around the period 2010–2020 (Supplementary Table 1).

City-level GII: The city-level GII was adapted from the Gender Inequality Index developed by the United Nations Development Program (UNDP), which compares men and women in health, empowerment (educational and political), and labor market dimensions at the country-level (United Nations Development Programme (UNDP), 2020). For the purposes of this study, we recreated the index at the city-level including only the labor market and empowerment dimensions since the health dimension includes ABR as an indicator. The index was constructed using city-level data from census measures and a manual search for mayoral elections in each country.

1. The labor market dimension assessed labor force participation, i.e., the percent of females and males aged 15+ in the labor force.
2. The empowerment dimension includes:
a. Education assessed by the percent of women and men aged 25 years or more with at least high school education
b. Government representation assessed by the percent of mayoral positions occupied by women and men. The percent of elected mayors who were women or men was assessed for elections conducted between the years 2010 and 2014 (except for Argentina where the period was from 2015 to 2017 and Peru where the year was 2015) by reviewing 1500 records of elected mayors. In Mexico and Argentina elections are not concurrent within the country, so we selected the latest election year for each jurisdiction within the period. In addition, some large SALURBAL cities included multiple jurisdictions, each of which elected a mayor. Of the 363 cities in the sample 176 had only 1 mayoral election and 187 had more than 1 mayoral election. . The number of elections in cities with >1 election had a median of 12 elections (range from 2 to 76). Among cities with >1 election the percent of elected women had a median of 0 (range 0–14%). Among cities with only one election 10.7% elected a woman. Given the highly zero inflated distribution of percent women mayors, when exploring this variable as a separate indicator, we dichotomized this indicator as the number of cities with at least one female mayor in the study period.

To construct the GII, we followed the formulas described by the UNDP (including political participation as percent mayoral positions held by men and women) excluding the health dimension (Supplementary material 1). A higher value of the index indicates a greater loss in potential human development due to inequality between females and males in these two dimensions. The index ranges from 0 (0% inequality), which indicates that women and men fare equally, to 1 (100% inequality) which indicates that one sex fares poorly in comparison to the other.

Using factor analysis, we created measures of women's achievements (WA scores) in three domains: 1) autonomy score, includes factors related to female labor force participation and formal-marriage for women ages 15–17, where a higher score signifies higher autonomy (ranges −4.9 to 3.3; 2) education score, includes factors related to women's secondary and university education, where a higher value indicates higher education among women (ranges −4.6 to 6.9); and 3) health care score, includes factors related to health care utilization for women such as the proportion of women 25–49 years old with pap smear and women 50–79 years old with mammogram. Higher values indicate better health access among women (ranges −3.7 to 4.1). This score is only available for four of the nine countries (Argentina, Brazil, Chile, and Mexico) and therefore analyses using this score are limited to 129 cities. More information on specific variables and the process to create these scores is described in supplementary material 2.

Analyses were adjusted for several city and sub-city-level covariates considered potential confounders based on previous research (Brahmbhatt et al., 2014; Rodriguez et al., 2017; Borrell et al., 2013; Braverman Bronstein et al., 2022) The city-level covariates were: population size (for 2014–2016), population growth (relative growth from 2010 to 2015) and homicide rates (for 2014–2016). Population indicators were estimated using country-specific population projections (Bilal et al., 2021), and homicide rates were used as a proxy for city-level violence, and were obtained from vital registration statistics (ICD10 codes: X85-Y09, Y871). The sub-city level covariates, were: living conditions (including indicators of sanitation, overcrowding, and adolescent school attendance) and population educational achievement (including indicators of secondary and university educational attainment). These scores were developed by the SALURBAL project using principal components analysis for previous studies, and have been found to be associated with several health outcomes including ABR and infant mortality (Ortigoza et al., 1136). Higher scores indicate better socioeconomic environment. The sub-city population educational achievement score was not included in analyses of WA education score or education-related indicators given its substantial conceptual overlap with the WA education score at the city level.

## 3 Statistical analysis

We examined the distribution of the GII and the WA scores by city within countries and estimated interclass correlation coefficients by fitting linear multilevel models with a random intercept for country to decompose variability between and within countries. In addition, we examined the distribution of the GII index and WA scores as well as the indicators used to create them, and the study covariates, by quartiles of sub-city ABR. We also visually explored associations between our exposures and outcome, finding no evidence of non-linear associations (Supplementary Fig. 1).

To assess the associations of the GII, the WA scores, and each of the indicators used to create them, with ABR, we fitted unadjusted and adjusted 3-level negative binomial models for each exposure of interest separately with random intercepts at the city and country level to account for the nesting structure. We created two sets of models: model 1 or unadjusted, including each exposure separately; and model 2 or adjusted, including each exposure separately but adjusting for all other city and sub-city-level covariates (as indicated above) (note that the whole population education score was not included in adjusted models for WA education score and education related indicators). All the predictors were used as continuous variables and standardized to a mean of zero and a standard deviation of one, except for political participation which was coded binary (1 representing cities with at least one female mayor). We also repeated all analyses restricted to the 129 cities for which the WA health care score was available in order to verify that associations were consistent with those observed in the full sample. All analyses were done using the statistical software Stata® version 16 and R® version 3.9.1.

## 4 Results

Table 1 shows the distribution of the GII, WA scores, indicators, and covariates for the full sample and by ABR quartiles. We found higher values of the GII- indicating more gender inequality- and lower values of the three WA scores -indicating lower autonomy, educational attainment, and health care access for women-in cities within the higher ABR quartiles. The mean percentage of men and women with high school education was lower in the highest quartiles of ABR. This pattern was also consistent for the mean percentage of women achieving university education. The mean percentage of women participating in the labor force was lower in the highest quartiles of ABR, while the inverse was seen among men. We also found a lower proportion of women having received a pap smear or mammogram in the highest quartiles of ABR (third and fourth) compared to the lowest quartiles (first and second). The opposite pattern was observed for the percent of women 15–17 who are married, which was higher at the highest quartiles of ABR. The percentage of cities with at least one female mayor was higher for cities in the second and third ABR quartiles compared to the first and fourth. Regarding other characteristics of cities and sub-cities, population size was smaller and population growth was larger in the highest ABR quartiles than in the lowest quartiles. City-level homicide rates were markedly lower in the lowest ABR quartile, but similar across the other three. Both sub-city socioeconomic indicators (living conditions and educational attainment) showed marked trends towards lower values in the highest ABR quartiles.

**Table 1:** Descriptive statistics of the city GII, woman's achievements scores, indicators, and covariates by quartiles of sub-city adolescent birth rates.

	Overall	First (Lowest)	Second	Third	Fourth (highest)	P value
ABR range (sub-city level)	(1.0–246.8)	(1.0–43.0)	(43.0–58.6)	(58.6–75.3)	(75.4–246.8)	
Sub-cities (N)	1403	351	351	351	350	
Cities (N)^a^	360	105	165	167	130	
**City-level scores (mean (sd))**						
WA autonomy score	0.03 (2.49)	0.83 (1.35)	0.55 (1.24)	−0.03 (1.23)	−0.64 (1.13)	<0.001
WA education score	0.03 (1.85)	2.08 (1.80)	0.79 (1.60)	0.17 (1.75)	−0.15 (1.87)	<0.001
WA health care access score^a^	0.16 (1.99)	1.12 (1.88)	1.13 (2.12)	0.17 (2.13)	−1.11 (1.50)	<0.001
Gender Inequality Index	0.27 (0.14)	0.23 (0.13)	0.27 (0.14)	0.29 (0.14)	0.29 (0.14)	<0.001
**City-level Indicators (mean(sd))**						
**Education**						
% Women 25 and older with high school	41.68 (12.24)	54.79 (13.28)	45.66 (10.39)	41.49 (10.61)	39.35 (11.32)	<0.001
% Men 25 and older with high school	41.75 (13.31)	57.73 (16.53)	46.32 (11.88)	42.13 (10.76)	40.59 (10.47)	<0.001
% Women 25 and older with university	15.73 (4.72)	18.39 (4.13)	15.93 (4.13)	14.64 (4.53)	13.97 (4.84)	<0.001
**Labor force**						
% Women 15 and older in the labor force	50.18 (8.43)	54.12 (6.70)	52.71 (7.42)	49.17 (8.15)	44.73 (6.33)	<0.001
% Men 15 and older in the labor force	76.32 (3.71)	76.09 (3.36)	76.42 (3.28)	76.76 (3.16)	77.32 (2.75)	<0.001
**Health care**						
% Women 50–79 with mammogram in the last 2 years^a^	42.24 (16.20)	51.86 (15.6)	51.57 (16.8)	43.23 (16.7)	33.45 (12.05)	<0.001
% Women 25–49 with pap smear in the last 3 years^b^	72.34 (8.26)	76.21 (6.57)	75.83 (8.95)	72.51 (9.12)	67.84 (6.68)	<0.001
**Political participation and child marriage**						
Cities with at least 1 female mayor (N, %)	83 (22.9)	32 (30.4)	52 (31.5)	48 (28.7)	36 (27.7)	0.893^c^
% Women 15-17 married/15–17 female pop	0.79 (1.18)	0.63 (0.88)	0.46 (0.64)	0.78 (1.02)	1.12 (1.25)	<0.001
**City and sub-city level Covariates (mean(sd))**						
Population size average (100,000 people) (city level)	24.31 (62.53)	129.69 (158.42)	125.79 (195.72)	102.45 (178.35)	113.16 (197.65)	0.185
Percent population growth (2010–2015) (city level)	6.45 (3.62)	6.37 (2.37)	5.69 (2.40)	6.70 (3.14)	6.93 (3.21)	<0.001
Homicide rates (per 100,000 people) (city level)	24.64 (21.25)	10.59 (10.56)	21.56 (17.67)	24.71 (18.98)	23.46 (19.51)	<0.001
Living conditions score (sub-city level)^d^	0.29 (2.42)	1.49 (1.75)	0.98 (1.83)	−0.34 (2.48)	−2.16 (2.67)	<0.001
Educational attainment score (sub-city level)^e^	−0.51 (1.03)	1.03 (1.99)	−0.23 (1.13)	−0.84 (1.10)	−1.28 (1.02)	<0.001

ABR: Adolescent birth rates, live births per 1000 women aged 15–19 years.

We present mean, standard deviations and p-values resulting from ANOVA test for all variables, otherwise specified.

Some cities have more than 1 sub-city and appear in more than 1 quartile.

a This variable is only available for 129 cities, 4 countries (28 cities in the first quartile; 60 cities in the second; 73 cities in the third; and 66 cities in the fourth).

b For Peru the age range is 30–49, this indicator is only available for 176 cities in 6 countries.

c Two-side chi-square test.

d SALURBAL living conditions score: proportion of households with piped water, overcrowding, and school attendance for people 15–17 years old.

e SALURBAL educational attainment score: proportion of people 25+ who completed secondary education or more and proportion of people 25+ who completed university education or more.

**Fig. 1:**
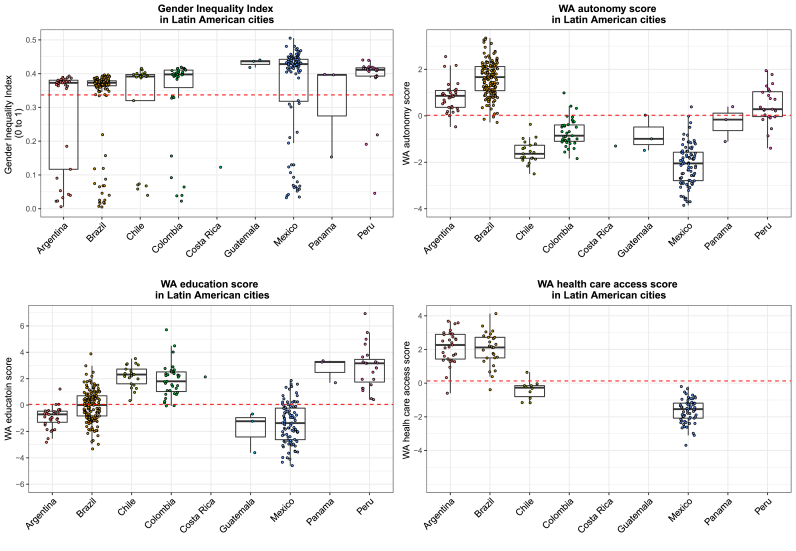
**Distribution of the Gender Inequality Index and Women Achievement (WA) scores by country**. Each dot represents a city within a country and the dotted line represent the overall mean value of each score. The intraclass correlation coefficients show that 73% of the variation in GII, 15% of the variation in WA health care score, 25% of the variation in WA education score, and 27% of the variation in WA autonomy score are between cities within countries.

The GII had the greatest variability across cities within countries but did not appear to vary much between countries (ICC 0.27). Within each country, the city GII showed a bimodal distribution, with some cities grouped with values close to zero and most cities with values around 0.4. This pattern is consistent across countries. The WA scores also vary within countries, but there is greater variation between countries, with the WA health care score having the highest between country variability (ICC WA autonomy 0.70; WA education 0.71; and WA health care 0.83). Cities in Mexico consistently had values below the overall mean of the WA scores and cities in Brazil, Panama, and Peru had values around or above the mean. Chile and Colombia had values below the mean for the WA autonomy score but above the mean for the WA education score (Fig. 1).

Table 2 shows associations of the GII index, WA scores, and their component indicators with ABR. We found associations of the GII, WA autonomy and WA health care scores with ABR after adjustment for other characteristics of the social environment at the city and sub-city level. Specifically, we found that the ABR was 4% higher for each 1 standard deviation (1-SD) higher GII (RR 1.04; 95%CI 1.01,1.06), 8% lower for each SD higher WA autonomy score (RR 0.92; 95%CI 0.86, 0.99), and 12% lower for each SD WA health care access score (RR 0.88; 95%CI 0.82,0.95). The WA education score also showed a significant inverse association with ABR when adjusted for covariates excluding the sub-city educational attainment score, with ABR 7% lower for each SD higher WA score 2 (RR 0.93; 95%CI 0.89,0.96); as expected, this association became weaker and was no longer statistically significant when the sub-city population educational attainment (for men and women combined) was added to the model (RR 0.98; 95%CI 0.95,1.02).

**Table 2:** Rate ratios of sub-city ABR associated with a 1-SD higher value of city-level scores and indicators before and after adjustment for city and sub-city level covariates (363 cities except where noted).

	Contrast	Unadjusted	Adjusted^a^
		RR (95%CI)	RR (95%CI)
**City Scores**			
WA autonomy score	1.36	**0.81 (0.77,0.86)**	**0.92 (0.86,0.99)**
WA education score	1.95	**0.85 (0.82,0.89)**	**0.93 (0.89,0.96)^e^**
WA health care score^b^	2.14	**0.83 (0.76,0.90)**	**0.88 (0.82,0.95)**
Gender Inequality Index	0.14	**1.04 (1.01,1.07)**	**1.04 (1.01,1.06)**
**City Indicators**			
**Education**			
% Women 25 and older with high school	12.88	**0.86 (0.81,0.92)**	**0.95 (0.90,1.00)**^e^
% Men 25 and older with high school	14.31	**0.82 (0.77,0.87)**	**0.91 (0.86,0.96)**^e^
% Women 25 and older with university	4.72	**0.87 (0.84,0.90)**	**0.93 (0.90,0.96)^e^**
**Labor force**			
% Women 15 and older in the labor force	8.04	**0.82 (0.78,0.86)**	**0.88 (0.85,0.92)**
% Men 15 and older in the labor force	3.18	0.98 (0.95,1.01)	0.99 (0.96,1.01)
**Health care**			
% Women 50–79 with mammogram in the last 2 years^b^	0.17	**0.83 (0.77,0.89)**	**0.87 (0.82,0.93)**
% Women 25–49 with pap smear in the last 3 years^c^	0.09	**0.89 (0.82,0.96)**	0.95 (0.89,1.01)
**Political participation and child marriage**			
Cities with at least 1 female elected mayor^d^	–	**0.93 (0.87,0.99)**	**0.94 (0.89,0.99)**
% Women 15-17 married/15–17 female pop	1	1.03 (0.99,1.07)	1.00 (0.97,1.03)

All variables were standardized to a mean of 0 and a standard deviation of 1, the association reflects the difference associated with a 1 standard deviation higher value (corresponding to the value in the Contrast column), unless otherwise specified. Each row corresponds to a separate model (exposures of interest are examined one at a time in separate models). Results come from a model of sub-cities nested in cities, with adjustment variables as defined below.

a Adjusted 1, models are adjusted for: population size, population growth, and homicide rates at a city level, and living conditions and educational attainment scores at sub-city level.

b This variable is only available for 129 cities, 4 countries.

c For Peru the age range is 30–49, this indicator is only available for 176 L1AD, 6 countries.

d Variable included as binary (0/1).

e The models for these variables exclude the sub-city population educational attainment score. The results when sub-city population educational attainment was included were as follows: WA education score (RR 0.98; 95%CI 0.95,1.02), % women 25 and older with high school (RR 1.03; 95%CI 0.98,1.09), % men 25 and older with high school (RR 0.98; 95%CI 0.93,1.03), % women 25 and older with university (RR 0.97; 95%CI 0.94,1.00).

Among the city-level indicators used to build the index and scores, percent of women and men 25+ with high school education and percent women 25+ with university education showed an inverse significant association with ABR (adjusted for covariates excluding sub-city population educational attainment). This association was stronger for men's educational attainment compared to women's (RR women with high school 0.95 95%CI 0.90–1.00; RR men with high school 0.91 95%CI 0.86–0.96; and RR women with university 0.93 95%CI 0.90–0.96, respectively). Fully adjusted models showed that each SD increase in female labor force participation was associated with 12% lower ABR (RR 0.88 95% CI 0.05,0.92). While the relationship for men was in the same direction, it was very weak and was not statistically significant. Both health care indicators showed an inverse association with ABR in unadjusted models, however, only the percent women 50–79 with mammogram remained significantly associated in fully adjusted models, with ABR being 13% lower for each SD higher percent women with mammogram (RR 0.87 95%CI 0.82,0.93). Cities that had at least one female mayor had 6% lower ABR compared to cities with no female mayors in fully adjusted models (RR 0.94 95%CI 0.89,0.99). The percent of women 15-17 married among the 15–17 population was not associated with ABR. Results for GII and WA autonomy and education scores, and all indicators remain similar when restricted to cities with data on WA health care score (Supplementary Table 2).

## 5 Discussion

Our study exploring gender inequalities, women's empowerment, and adolescent birth rates in 363 Latin American cities, had two key findings. First, we found large heterogeneity in the distribution of the GII and the WA scores across cities within countries, indicating that national measures of gender inequality obscure wide sub-national variability. Second, we found that lower levels of gender inequality and higher levels of women's empowerment (specifically the autonomy and health care access scores) were associated with lower adolescent birth rates, even after adjusting for other city and sub-city-level covariates. When examining the individual indicators that were included in the scores and index, we found that a higher percent of women in the labor force, a higher percent of women and men with high school and women with university education, a higher percent of women 50–79 with a mammogram in the last two years and having at least one female mayor in the city were associated with lower ABR after adjusting for other covariates.

Despite the implementation of policies to meet family planning needs and universal health care coverage, in LA ABR have been decreasing slowly; if this trend continues LA will not achieve the Sustainable Development Goals (SDG) on adolescent pregnancy by 2030 (Rodriguez et al., 2017). The region is characterized by high urbanization, heterogenous social urban environments and important social, economic, and gender inequalities (Ortigoza et al., 2021). Conservative politics and the blockage of initiatives to introduce gender related content in schools combined with restrictive laws and policies regarding sexual and reproductive health have been proposed as mechanisms for the slow declines of ABR within the region (Caffe et al., 2017; Rodriguez et al., 2017). Evidence suggests that empowering young women in the educational, community, economic, and health spheres helps reduce adolescent pregnancy (Nkhoma et al., 2020), however, these factors have not yet been systematically examined at the city-level. We found large heterogeneities between cities within countries regarding gender inequality and women's empowerment, and important associations with ABR. This heterogeneity, while expected, stresses the need for more subnational studies and highlights the opportunity to implement local policies aimed at reducing gender gaps in cities to improve the overall situation of women.

Adolescent pregnancy reflects multiple vulnerabilities and deficiencies in children and adolescent's rights (Fabiana Reina and Castelo-Branco, 2018). Inequalities in the economic, educational, social, and gender spheres are both determinants and consequences of adolescent pregnancy (Pan American Health Organization (PAHO) and United Nations Population Fund (UNFPA), 2016). Marked increases in gender violence in several countries such as Mexico and Colombia highlight the increased vulnerability and lack of protection for women within the region (Müller, 2018; Drewry and Garcés-Palacio, 2020). The restrictive policies that reinforce gender roles and limit women's access to education, reproductive health, and autonomy within the region also have important variations within countries (Näslund-Hadley and Binstock, 2010). For example, in Mexico, despite important efforts to implement comprehensive sex education and include gender related content in school curriculum at a national level, some state governments have resisted these changes (Rodriguez et al., 2017; de Castro et al., 2018). The GII heterogeneities found within countries underscore the need for local policies tailored to each city's context. Our results showing higher levels of the GII (reflecting higher inequality) are associated with higher ABR, reinforce the need to prioritize city policies to reduce gender inequalities as a way to support further reductions in adolescent pregnancy rates.

We found important inverse associations between the three WA scores and ABR. This is consistent with other research showing that economic empowerment of women and policies favoring education and health care for women are associated with a reduction of adolescent pregnancies (Nkhoma et al., 2020). Economic empowerment of girls and women through employment opportunities and cash-exchange programs have been shown to reduce economic and social constraints that often lead to unhealthy sexual behaviors (Reed et al., 2018). In our study, the WA autonomy score, which reflects higher percent of women in the labor force and a lower percent of formal child marriage was associated with lower ABR, consistent with prior work.(Chung et al., 2018)A recent study investigating the impact of social determinants on maternal and child health outcomes in 47 low-and middle-income countries found that educational attainment and child marriage would have the largest impact on reducing ABR if universal completion of secondary school for women and elimination of child marriage were achieved (Kidman and Heymann, 2018). When analyzing the percent formal child marriage indicator alone we found no association, yet this might be explained by the fact that our indicator included only women 15–17 years old and excluded cohabitation, which is common in Latin American contexts and has been found to be associated with adolescent pregnancy (Taylor et al., 2019).

LA countries have greatly increased educational opportunities, in some cases resulting in higher school attendance rates for girls compared to boys (United Nations Educational Scientific and Cultural Organization (UNESCO), 2020). Higher educational attainment as well as school attendance have been consistently associated with lower ABR across the world (Nkhoma et al., 2020). In our study we found that higher levels of the WA education score, as well as all educational indicators, were associated with lower ABR. Interestingly the most strongly associated indicator was the percent of men 25 year and older with at least high school education. This could relate to the fact that women with more educated male partners are more likely to use contraception (Adjiwanou et al., 2018), highlighting the need to include men when designing adolescent pregnancy prevention interventions (IPPF UNFPA, 2017; International Planned Parenthood Federation, 2010).

We also found that higher city levels of reproductive health care (WA health care score) were strongly associated with lower ABR, supporting the evidence from national and individual studies regarding the importance of health care access and utilization to reduce ABR (Caffe et al., 2017; Dongarwar and Salihu, 2019). In our analyses cities with at least one female mayor had lower ABR, although the association was weak, possibly due to the limitations of our indicator. In addition, our measure captures participation of women in government but not ideological or political orientation (progressive or conservative) which may add to or interact with female representation. The mechanisms for this association might be indirect, yet, prior work has found that female participation in government, is associated with implementation of policies targeted at improving education and work conditions for women, in addition to focusing on improving maternal and child living conditions and health outcomes (Gideon and Ramm, 2020). These findings point towards the importance of local policies supporting female political participation, employment, education, and health-care at a city-level as additional strategies to reduce ABR.

There are some limitations to our analyses. The GII, is an attempt to replicate the UNDP national level GII at a city-level, but it lacks the health component that would make it comparable to that index, given that this component includes adolescent birth rates as an indicator. Also, the use of percent mayoral positions as opposed to the percent of parliament seats in the creation of the index resulted in a binomial distribution (0 or 1) for cities with only one sub-city making our measure zero inflated and resulting in a bimodal distribution for the GII. Still, it provides a useful measure of gender inequality within the empowerment and labor market spheres. This measure is highly correlated with the complete city-level GII (r = 0.8) and when adjusted models are fitted with this version, they yield similar results (OR 1.06; 95%CI 1.04,1.09). Furthermore, when estimating the correlation of the country-average city-level GII with the national GII estimated by UNDP we obtain high correlations for both versions of the index (r = 0.8901 for the complete and 0.6083 for the GII excluding the health component) suggesting comparability between the city-level GII we developed and the UNDP measure.

Child marriage has been consistently associated with higher ABR across the globe, however, the definition most commonly used generally includes both formal and informal marriage for any person before the age of 18 (UNICEF. Child marriage, 2020); for data comparability across countries we only included formal marriage for women 15 to 17 years-old, which results in a much lower prevalence and could reduce variability, possibly explaining the lack of associations of this variable with ABR in our analyses. We adjusted all models for a series of social environment variables at the city and sub-city level which may be confounders. Adjustment variables were not strongly correlated with our main exposures of interest (Supplementary Tables 3 and 4). However the inclusion of the educational attainment score at a sub-city level strongly affected the association of ABR with the city level educational scores, suggesting that the more proximal sub-city measures of education may be more important to ABR than the city level measures. The health care measures were only available for a limited number of cities. When we examined other associations in the smaller subset we found similar patterns to those described for the full sample. (Supplementary Table 2). We acknowledge that these health care indicators might not completely measure reproductive health care for adolescent women, nevertheless they measure female utilization of preventive health care which is an important component of reproductive health. Ideally the inclusion of indicators reflecting adolescent use of reproductive health care services would be better, however, disaggregated data on this regard is lacking in the region. Although we attempted to adjust for potential confounders, residual confounding by imperfectly measured or omitted variables cannot be ruled out. Last, our study is cross-sectional, and cannot untangle the potential reverse associations between ABR and, for example, educational achievement. Given the role adolescent pregnancies play in reducing educational opportunities for women in some settings, it is also plausible that higher rates of ABR are driving lower educational achievement.

While there is some evidence regarding gender inequality and adolescent birth rates (Nkhoma et al., 2020; Kidman and Heymann, 2018), this is the first attempt to look at city levels of gender inequality and women's empowerment and their association with ABR in LA. We included information on 363 cities which represent urban areas within 9 countries in LA. The use of multilevel modeling allowed us to include different variables at different levels by accounting for the clustering effects of sub-cities within cities within countries. Given the large heterogeneities found in urban environments in LA, looking at these associations at a city and sub-city level is extremely valuable to inform local policies and move forward the adolescent pregnancy prevention agenda.

Our findings support the need to reduce gender gaps and inequality in the economic, educational, health care, and political spheres to decrease ABR within the region. It is imperative to support but also to act beyond comprehensive sex education and contraceptive access, to achieve the adolescent pregnancy 2030 SDG in the LA region. Despite positive movements towards implementing policies aimed at protecting women such as decriminalizing abortion, there are still important structural factors and cultural norms in the region that prevent young women from achieving social and economic independence (Rodríguez Ribas, 2021). Promoting policies that incentivize women to achieve higher education levels and generate more opportunities for female employment and health care utilization could be an effective way to tackle adolescent pregnancy. In addition, increasing the inclusion of women in the local political environment as mayors, governors or other political representatives could change the perception of gender roles and impact decision making processes in young women further decreasing ABR, in addition to directly influencing policy changes critical to improving gender equality.

## Author credits

Ana V. Diez Roux: funding acquisition, supervisión; Tonatiuh Barrientos Gutierrez: conceptualization, Writing - Review & Editing; Ariela Braverman-Bronstein: methodology, formal analysis, Writing - Original Draft; Ana F Ortigoza and Desirèe Vidaña-Pérez: investigation and Writing - Original Draft. Laura Baldovino Chiquillo, Usama Bilal, Amélia Augusta de Lima Friche, Francisco Diez-Canseco, Julie Maslowsky, Alejandra Vives V.: Writing - Review & Editing. All authors reviewed and approved the final version of the manuscript.

## Funding

The Salud Urbana en América Latina (SALURBAL)/Urban Health in Latin America project is funded by the 10.13039/100010269Wellcome Trust [205177/Z/16/Z]. UB was also supported by Office of the Director of the National Institutes of Health under award number DP5OD26429

## Role of the funding source

The funding source was not involved in data collection, analysis or interpretation of data, manuscript writing, or the decision to submit the manuscript.

## Declaration of competing interest

None.

## Data Availability

The SALURBAL project welcomes queries from anyone interested in learning more about its dataset and potential access to data. To learn more visit https://drexel.edu/lac/or contact http://salurbal@drexel.edu.
