# Supplementary material for: Gender inequality, women's empowerment, and adolescent birth rates in 363 Latin American cities
Source: Soc Sci Med. 2023 Jan;317:115566. doi: 10.1016/j.socscimed.2022.115566 (PMC7613905; doi:10.1016/j.socscimed.2022.115566)
Supplement: Multimedia component 1 [file mmc1.docx]

**Supplementary table 1.** Data sources by year and country.

| Country | Census | Political Participation | Health Survey | Vital registration |
| --- | --- | --- | --- | --- |
| Argentina | 2010 | 2015, 2017 | 2013 | 2014-2016 |
| Brazil | 2010 | 2012 | 2013 | 2014-2016 |
| Chile | 2017* | 2012 | 2010 | 2014-2016 |
| Colombia | 2018 | 2011 | NA | 2014-2016 |
| Costa Rica | 2011 | 2010 | NA | 2014-2016 |
| Guatemala | 2018 | 2011 | 2015 | 2014-2016 |
| Mexico | 2010 | 2010-2014 | 2012 | 2014-2016 |
| Panama | 2010 | 2014 | NA | 2014-2016 |
| Peru | 2017 | 2015 | 2016 | 2014-2016 |

*Child marriage data comes from the 2002 census

**Supplementary material 1:** Formulas adapted from the United Nations Development Program for the estimation of the Gender Inequality Index

Step 1.- Create summary indices for males and females separately aggregating across dimensions using geometric means

For women:

$$G_{f}=\sqrt[2]{\left( \sqrt{PP_{f}*HS_{f}}*{LFP}_{f} \right)}$$

For men:

$$G_{m}=\sqrt[2]{\left( \sqrt{PP_{m}*HS_{m}}*{LFP}_{m} \right)}$$

Where PP is political participation (% mayors), HS is population aged 25+ with at least high school education, and LPF is population aged 15+ in the labor force. Subindices refer to males (m) and females (f).

Step 2: Take the harmonic mean of the female and male indices.

$$HARM (G_{f}, G_{m})=\left( \frac{\left( Gf^{-1}+Gm^{-1} \right)}{2} \right)^{-1}$$

Step 3: Calculate the geometric mean of the arithmetic means for each indicator.

$$G_{f,m}=\sqrt[2]{\bar{EMP}*\bar{LFPR}}$$

$$\bar{EMP}=\frac{\left( \sqrt{PP_{f}*HS_{f}}+ \sqrt{PP_{m}*HS_{m}} \right)}{2}$$

$$\bar{LFPR}=\frac{\left( LFP_{f}+LFP_{m} \right)}{2}$$

Step 4: Compare HARM (G_f_, G_m_) to G_f,m_ to derive the GII. When HARM (G_f_, G_m_) is equal to G_f,m_, there is 100% inequality.

$$GII=1-\left( \frac{HARM (G_{f}, G_{m})}{G_{f,m}} \right)$$

*For justification of the specific formulas see: United Nations Development Programme (UNDP). Technical Notes Calculating the Human Development Indices-Graphical Presentation Inequality-Adjusted Human Development Index (IHDI) Knowledge Human Development Index (HDI) Long and Healthy Life A Decent Standard of Living Human Development Index (HDI) Kno.; 2020.*

**Supplementary material 2:** Process for creating the Women’s Achievements scores

These scores were derived from factor analysis of 6 indicators: % of women 15+ in the labor force; % of women aged 15-17 who are married among the 15-17 female population; % women ages 25+ with at least high school education; % women ages 25+ with at least university education; % of women ages 25-49 with a pap smear in the last 3 years; and the % of women 50-79 with a mammogram in the last 2 years. After varimax rotation, the factor analysis yielded two factors with an eigenvalue greater than 1: one grouping the employment, marriage, and health indicators, and the other grouping the education indicators. Given that data was not available for the health indicators in all cities, we separated the health indicators from the first factor into a third factor. This resulted in the creation of three WA scores. A score for each factor was created by summing Z scores for each indicator that loaded on the factor ≥ 0.5. The three scores are described below.

- WA employment score: includes factors related to employment and marriage; % of women 15+ in the labor force and % women 15-17 who are married among the 15-17 female population. For the construction of the score, we reverse coded the marriage indicator since their correlation was negative. A higher score signifies higher economic autonomy.
- WA education score: includes factors related to women’s education; % women ages 25+ with at least high school education; % women ages 25+ with at least university education. A higher value indicates higher education among women.
- WA health care access score: includes factors related to health care access for women. The % of women ages 25-49 with a pap smear in the last 3 years and the % of women 50-79 with a mammogram in the last 2 years. This score is only available for a limited number of countries (Argentina, Brazil, Chile, and Mexico). The data for these indicators was retrieved from national health surveys, and the prevalence at a city-level result from model based standardized smoothed estimates. These measures were used as indicators for health care access for women. A higher value indicates better health access among women.


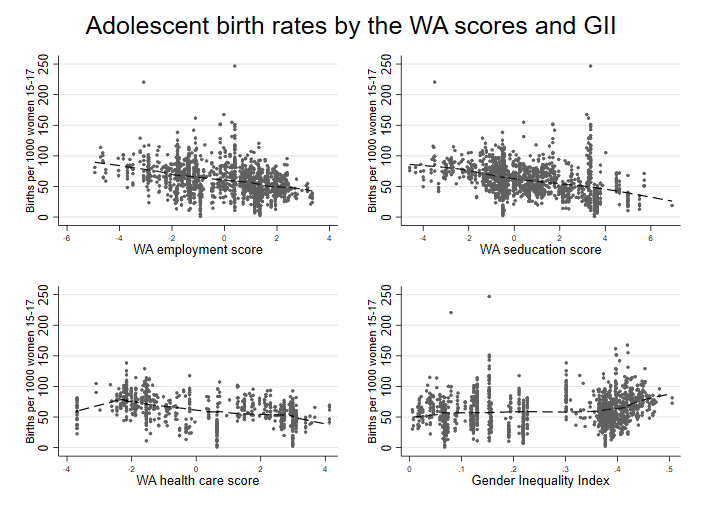


**Supplementary figure 1-** Scatter plots with lowess fit for the crude association of adolescent birthrates and the Women’s Achievements (WA) scores and Gender Inequality Index

**Supplementary table 2 Rate ratios of ABR associated with a 1SD higher value of each indicator before and after adjustment for 129 cities with complete data on WA health care access score**

|  |  | **Unadjsuted** | **Adjusted^1^** | **Adjusted^2^** |
| --- | --- | --- | --- | --- |
|  | **Contrast** | **RR (95%CI)** | **RR (95%CI)** | **RR (95%CI)** |
| **Scores** |  |  |  |  |
| WA employment score | 2.42 | **0.88 (0.81,0.95)** | **0.92 (0.86,0.99)** | 0.96 (0.88,1.04) |
| WA education score | 1.43 | **0.84 (0.78,0.90)** | 0.96 (0.90,1.02) | **0.90 (0.84,0.96)** |
| WA health care access score | 2.14 | **0.83 (0.76,0.90)** | **0.88 (0.81,0.95)** | 0.94 (0.86,1.03) |
| Gender Inequality Index | 0.1 | **1.06 (1.02,1.09)** | **1.06 (1.03,1.10)** | **1.06 (1.02,1.09)** |
| **Education** |  |  |  |  |
| % Women 25 and older with high school | 9.47 | **0.80 (0.73,0.89)** | 0.98 (0.89,1.08) | **0.89 (0.81,0.97)** |
| % Men 25 and older with high school | 9.24 | **0.77 (0.70,0.85)** | 0.94 (0.85,1.05) | **0.84 (0.77,0.92)** |
| % Women 25 and older with university | 3.46 | **0.87 (0.82,0.92)** | 0.96 (0.91,1.00) | **0.92 (0.87,0.97)** |
| **Labor Force** |  |  |  |  |
| % Women 15 and older in the labor force | 8.43 | **0.85 (0.79,0.91)** | **0.89 (0.84,0.95)** | **0.90 (0.84,0.97)** |
| % Men 15 and older in the labor force | 3.01 | 0.98 (0.93,1.02) | 0.97 (0.93,1.01) | 0.98 (0.94,1.02) |
| **Health care** |  |  |  |  |
| % Women 50-79 with mammogram in the last 2 years | 0.17 | **0.84 (0.78,0.90)** | **0.87 (0.82,0.93)** | 0.94 (0.87,1.01) |
| % Women 25-49 with pap smear in the last 3 years | 0.09 | **0.92 (0.85,0.98)** | 0.95 (0.89,1.01) | 0.98 (0.91,1.04) |
| **Political participation and child marriage** |  |  |  |  |
| % Cities with at least 1 female elected mayor^3^ | - | **0.90 (0.83,0.97)** | **0.88 (0.83,0.95)** | 0.98 (0.95,1.02) |
| % Cities with at least 1 male elected mayor^3^ | - | 0.97 (0.72,1.31) | 0.92 (0.71,1.22) | 1.02 (0.99,1.05) |
| % Women 15-17 married / 15-17 female pop | 1.01 | 1.01 (0.97,1.06) | 1.00 (0.96,1.04) | 0.97 (0.93,1.02) |

All variables were standardized to a mean of 0 and a standard deviation of 1, the association reflects the difference associated with a 1 standard deviation higher value (corresponding to the value in the Contrast column), unless otherwise specified. Each row corresponds to a separate exposure of interest modeled. Results come from a model of sub-cities nested in cities, with adjustment variables as defined below.

1. Adjusted 1, models are adjusted for: population size, population growth, and homicide rates at a city level, and living conditions and educational attainment scores at sub-city level.
2. Adjusted 2, models are adjusted for: population size, population growth, and homicide rates at a city level, and living conditions at sub-city level.
3. Variable included as binary (0/1)

**Supplementary table 3: Correlation matrix for the women’s achievements scores 1 and 2, gender inequality index and city and sub-city social environment variables adjustment variables (363 cities)**

|  | **WA**  **employment** | **WA**  **education** | **GII** | **Population**  **size** | **Population**  **growth** | **Living**  **conditions** | **Educational**  **attainment** | **Homicide rates** |
| --- | --- | --- | --- | --- | --- | --- | --- | --- |
| WA employment | 1 |  |  |  |  |  |  |  |
| WA education | 0.2939 | 1 |  |  |  |  |  |  |
| GII | -0.0173 | -0.2331 | 1 |  |  |  |  |  |
| Population size | 0.0646 | -0.0025 | -0.2526 | 1 |  |  |  |  |
| Population growth | -0.0623 | 0.0557 | 0.1003 | -0.2616 | 1 |  |  |  |
| Living conditions | 0.5452 | 0.2265 | -0.1107 | 0.0149 | -0.1718 | 1 |  |  |
| Educational attainment | 0.2003 | 0.4525 | -0.0402 | 0.0468 | 0.0998 | 0.4504 | 1 |  |
| Homicide rates | 0.1412 | -0.2504 | 0.0328 | -0.1179 | -0.1694 | 0.095 | -0.2135 | 1 |

**Supplementary table 4: Correlation matrix for the women’s achievements scores, gender inequality index and city and sub-city social environment variables adjustment variables (129 cities)**

|  | **WA**  **Employment** | **WA**  **Education** | **WA**  **Health care** | **GII** | **Population**  **size** | **Population**  **growth** | **Living**  **conditions** | **Educational**  **attainment** | **Homicide**  **rates** |
| --- | --- | --- | --- | --- | --- | --- | --- | --- | --- |
| WA employment | 1 |  |  |  |  |  |  |  |  |
| WA education | -0.1059 | 1 |  |  |  |  |  |  |  |
| WA health care | -0.4024 | 0.3157 | 1 |  |  |  |  |  |  |
| GII | -0.086 | 0.8617 | 0.3052 | 1 |  |  |  |  |  |
| Population size | 0.3032 | -0.2353 | -0.1385 | -0.2529 | 1 |  |  |  |  |
| Population growth | -0.2265 | 0.1573 | 0.0098 | 0.1556 | -0.4733 | 1 |  |  |  |
| Living conditions | -0.0871 | 0.6331 | 0.4365 | 0.7084 | -0.2515 | 0.047 | 1 |  |  |
| Educational attainment | 0.0344 | 0.1034 | 0.1732 | 0.1203 | -0.0007 | 0.0823 | 0.5195 | 1 |  |
| Homicide rates | -0.0172 | 0.3183 | 0.0094 | 0.1981 | -0.2129 | -0.1767 | 0.1167 | -0.0766 | 1 |
